# Mapping of *HKT1;5* Gene in Barley Using GWAS Approach and Its Implication in Salt Tolerance Mechanism

**DOI:** 10.3389/fpls.2018.00156

**Published:** 2018-02-19

**Authors:** Khaled M. Hazzouri, Basel Khraiwesh, Khaled M. A. Amiri, Duke Pauli, Tom Blake, Mohammad Shahid, Sangeeta K. Mullath, David Nelson, Alain L. Mansour, Kourosh Salehi-Ashtiani, Michael Purugganan, Khaled Masmoudi

**Affiliations:** ^1^Khalifa Center for Genetic Engineering and Biotechnology, United Arab Emirates University, Al Ain, United Arab Emirates; ^2^Center for Genomics and Systems Biology, New York University of Abu Dhabi, Abu Dhabi, United Arab Emirates; ^3^Laboratory of Algal and Systems Biology, New York University of Abu Dhabi, Abu Dhabi, United Arab Emirates; ^4^Department of Biology, College of Science, United Arab Emirates University, Al Ain, United Arab Emirates; ^5^Plant Breeding and Genetics, School of Integrative Plant Science, Cornell University, Ithaca, NY, United States; ^6^Department of Plant Sciences and Plant Pathology, Montana State University, Bozeman, MT, United States; ^7^International Center for Biosaline Agriculture, Dubai, United Arab Emirates; ^8^Department of Arid Land Agriculture, College of Food and Agriculture, United Arab Emirates University, Al Ain, United Arab Emirates; ^9^Date Palm Tissue Culture, United Arab Emirates University, Al Ain, United Arab Emirates

**Keywords:** GWAS, barley, salinity tolerance, *HKT1;5* gene, sodium transport

## Abstract

Sodium (Na^+^) accumulation in the cytosol will result in ion homeostasis imbalance and toxicity of transpiring leaves. Studies of salinity tolerance in the diploid wheat ancestor *Triticum monococcum* showed that *HKT1;5*-like gene was a major gene in the QTL for salt tolerance, named *Nax2*. In the present study, we were interested in investigating the molecular mechanisms underpinning the role of the *HKT1;5* gene in salt tolerance in barley (*Hordeum vulgare*). A USDA mini-core collection of 2,671 barley lines, part of a field trial was screened for salinity tolerance, and a Genome Wide Association Study (GWAS) was performed. Our results showed important SNPs that are correlated with salt tolerance that mapped to a region where *HKT1;5* ion transporter located on chromosome four. Furthermore, sodium (Na^+^) and potassium (K^+^) content analysis revealed that tolerant lines accumulate more sodium in roots and leaf sheaths, than in the sensitive ones. In contrast, sodium concentration was reduced in leaf blades of the tolerant lines under salt stress. In the absence of NaCl, the concentration of Na^+^ and K^+^ were the same in the roots, leaf sheaths and leaf blades between the tolerant and the sensitive lines. In order to study the molecular mechanism behind that, alleles of the *HKT1;5* gene from five tolerant and five sensitive barley lines were cloned and sequenced. Sequence analysis did not show the presence of any polymorphism that distinguishes between the tolerant and sensitive alleles. Our real-time RT-PCR experiments, showed that the expression of *HKT1;5* gene in roots of the tolerant line was significantly induced after challenging the plants with salt stress. In contrast, in leaf sheaths the expression was decreased after salt treatment. In sensitive lines, there was no difference in the expression of *HKT1;5* gene in leaf sheath under control and saline conditions, while a slight increase in the expression was observed in roots after salt treatment. These results provide stronger evidence that *HKT1;5* gene in barley play a key role in withdrawing Na^+^ from the xylem and therefore reducing its transport to leaves. Given all that, these data support the hypothesis that *HKT1;5* gene is responsible for Na^+^ unloading to the xylem and controlling its distribution in the shoots, which provide new insight into the understanding of this QTL for salinity tolerance in barley.

## Introduction

The world's land area affected by salinized soil and water is approximately 7%, and according to the FAO Land and Plant Nutrition Management service, most of the world's land is not cultivated, but a significant proportion of irrigated land is salt-affected. In arid and semi-arid regions, soil salinization is a major threat to agriculture, where water scarcity and inadequate irrigation management will severely reduce crop yield (FAO, 2008). Accumulation of sodium in the cytosol of plants is toxic due to imbalance of ions in the transpiring leaves. There are two major mechanisms of salinity stress at the whole plant level that are advanced so far: a rapid and early osmotic stress which reduces shoot growth, and a slower accumulating ionic stress which accelerates senescence of older leaves (Sahi et al., 2006; Munns and Tester, 2008). Osmotic stress affects the plant's water relations due to reduced availability of water from the soil solution (Munns, 2005), which in turn disturbs the growth of the plant by reducing cell expansion and elongation rates. This mechanism will lead to smaller and thicker leaves, reducing photosynthesis by stomatal closure, and limiting water uptake (Fricke et al., 2004). To minimize the toxic effects of ionic Na^+^ stress, plants employ different mechanisms to adapt and tolerate saline conditions. The most important ones to minimize the harmful effects of ionic Na^+^ stress include immediate Na^+^ exclusion from uptake, limit of xylem Na^+^ loading and/or retranslocation from the shoot; efficient compartmentalization of Na^+^ mainly into vacuoles, cytosolic K^+^ homeostasis and preservation in root and mesophyll cells, efficient osmotic adjustment by a decrease of water loss and an increase of water uptake, and last but not least a ROS detoxification (Blumwald, 2000; Zhu, 2003; Munns and Tester, 2008). To tolerate salinity, plants deploy a variety of traits to control the function and development of the cell that relies on signal perception, signal integration and processing. Therefore, adaptation to salinity stress is a quantitative character, which is controlled by different genetic pathways, where multiple genes are implicated in salinity tolerance (DeRose-Wilson and Gaut, 2011).

Barley (*Hordeum vulgare*) is the major and most salt tolerant cereal crop worldwide (Munns and Tester, 2008). It can tolerate up to 250 mM NaCl (equivalent to 40% seawater), beyond which the survival rates drop drastically. Cultivated barley, its wild ancestor (*Hordeum vulgare subp. Spontaneum*) and domesticated barley are originated from the Fertile Crescent and Tibet (Kilian et al., 2006; Dai et al., 2012). Genetic diversity and adaptation of barley to extreme conditions resulted in a rich pool of genetic variation (Nevo and Chen, 2010). However, breeding efforts to select high yield and stability genotype of barley plants in marginal environment have met with limited success (Flowers and Flowers, 2005). Indeed, modern cultivated barely varieties share 15–40% of all alleles within the barley gene pool, which indicate that only a small fraction of barley genetic resource has been used to improve salinity tolerance (Long et al., 2013). Salinity tolerance in plants is under complex polygenic trait controlled by numerous quantitative trait loci (QTLs) and several genes have been proposed to be involved (Flowers, 2004). High-Affinity K^+^ Transporter (*HKT*) genes encode Na^+^ and/or K^+^ transport systems, active at the plasma membrane (Almeida et al., 2013; Véry et al., 2014). While weakly represented in dicot species genomes (e.g., one single HKT gene in Arabidopsis and poplar), the *HKT* family comprises more members displaying a large functional diversity in monocots, including cereals. For instance, rice (*Oryza sativa*) possesses 9 *HKT* genes (Garciadeblás et al., 2003), and barley (*Hordeum vulgare*) and wheat (*Triticum aestivum*) have been deduced from southern blot analyses to possess 5 to 11 *HKT* genes per genome, respectively (Huang et al., 2008). Based on phylogenetic and functional analyses, plant *HKT* genes have been divided into two subfamilies (Platten et al., 2006). Subfamily 1 *HKT* (present in all higher plant species) encode Na^+^-selective transporters, while subfamily 2 ones (monocot specific) encodes systems permeable to both Na^+^ and K^+^ (Jabnoune et al., 2009; Munns et al., 2012; Sassi et al., 2012; Ben Amar et al., 2014; Suzuki et al., 2016).

In durum wheat, two QTLs (*Nax1* and *Nax2*) were found to be involved in salinity tolerance. The *Nax2* region corresponds to the *HKT1;5* locus, which encodes a selective Na^+^ transporter. The *Nax2* locus on the 5AL chromosome is homologous to *Kna1* located on chromosome 4DL carrying the major QTL for Na^+^ exclusion in common wheat (Byrt et al., 2007; James et al., 2011; Arzani and Ashraf, 2016). Moreover, *HKT* genes shown to be associated to QTLs of salt tolerance belong to subfamily 1. They have been shown to play crucial roles in salinity tolerance in different plant species (Munns et al., 2012; Asins et al., 2013, 115; Arzani and Ashraf, 2016).

A wide range of physiological and agronomic traits was used as selection criteria to map QTL for salinity tolerance in barley. Among these, we can enumerate plant survival (Zhou et al., 2012; Fan et al., 2015), stomatal size, frequency, and photosynthesis parameters (Liu et al., 2017), yield and agronomic traits (Xue et al., 2009), seed germination and seedling growth stage (Witzel et al., 2010; Ahmadi-Ochtapeh et al., 2015), Na^+^ exclusion (Shavrukov et al., 2010), tissue ion content (Xue et al., 2009), water soluble carbohydrate and chlorophyll content (Siahsar and Narouei, 2010). In previous study, Salinity tolerance in barely was assessed through a combination of plant survival and leaf wilting (Zhou et al., 2012; Fan et al., 2015). These two major symptoms caused by salt stress had been used for evaluating salinity tolerance of barley through QTL mapping.

To uncover and elucidate the genetic basis of complex agronomic traits, genome-wide association studies (GWAS) have been increasingly used. To elucidate the genetic basis of plant height and inflorescence architecture in sorghum, GWAS was performed using genome-wide map of SNP variation that allowed to map several classical loci of plant height, candidate genes for inflorescence architecture (Morris et al., 2013). In rice, GWAS was implemented to identify loci controlling salinity tolerance. Depending on 6,000 SNPs in many stress-responsive genes, Infinium high-throughput assay was used to genotype 220 rice accessions. In addition to *saltol*, a major QTL, identified to control salinity tolerance at seedling stage, GWAS peaks representing new QTLs was found on chromosome 4, 6, and 7 (Kumar et al., 2015). In wheat, the analysis of the *Nax2*, which corresponds to *TmHKT1;5*-type gene, suggests that this gene display distinctive expression pattern in roots.

Here, we present a genome-wide association study (GWAS) in the USDA barley core collection. This population comprising 2,671 lines collected worldwide, selected to represent the entire 30,000 lines of the USDA collection, was evaluated for different agronomic characters performance and yield in the field in order to identify loci associated with salinity tolerance using single nucleotide polymorphism (SNP) data and to study the molecular mechanism underpinning the role of the *HKT1;5* ion transporter gene in slat tolerance in barley. Real time PCR experiments allowed to compare the expression patterns of *HKT1;5* gene in roots, leaves and leaf sheaths in tolerant and sensitive barley lines.

## Materials and methods

### Barley germplasm

**Figure 1:**
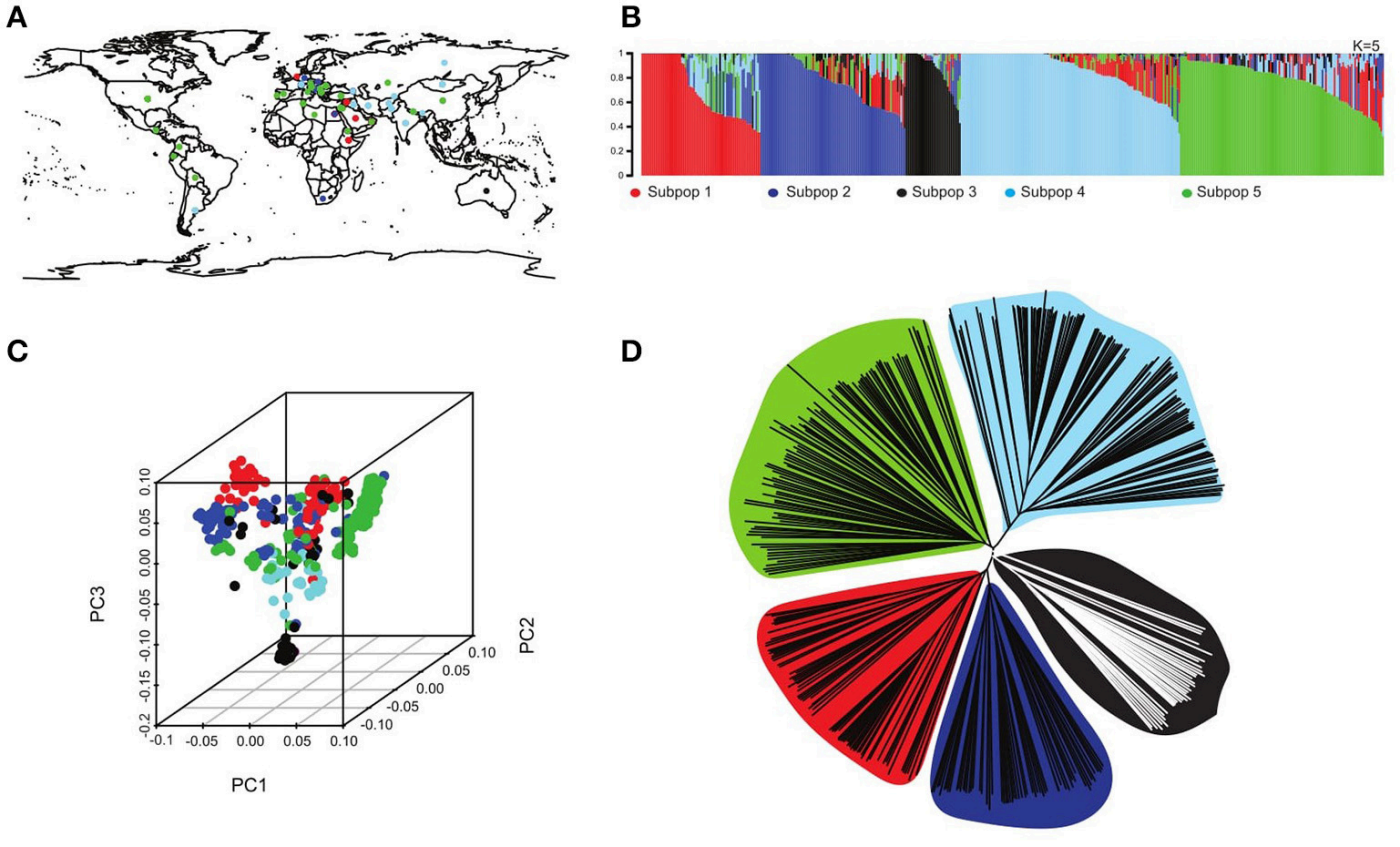
Population structure in the USDA core collection. **(A)** Geographical distribution of the different lines represented in the core collection, colors refer to the five different subpopulations. **(B)** Population structure run using the software STRUCTURE (Pritchard et al., 2000) of the 2761 accession depicting five subpopulations (*K* = 5) each represented with a different color with shared colors represent admixture group. **(C)** 3D principal component diagram showing the five subpopulations in different colors clustering consistent with the neighboring joining tree. **(D)** A neighboring joining tree showing the five different subpopulations.

A total of 2,671 barley accessions collected worldwide, selected to represent the entire 30,000 lines of the USDA barley collection (Figure 1A), was evaluated for salinity response to map the locations of genes contributing to low flag leaf Na^+^/K^+^ ratio under saline production environment using association analysis.

### Field trials and evaluation of salinity tolerance

Field trial was implemented at the International Center for Biosaline Agriculture (ICBA), Dubai, United Arab Emirates (25° 05′ 40.8″ N 55° 23′ 23. 5″ E), from November 2012 to May 2013.

The surface soil texture at ICBA field experimental station is composed of 98% sand, 1% silt, and 1% clay. The native soil is non saline, where the electrical conductivity of its saturated extract is 1.2 dS m^−1^). It is moderately alkaline (pH 8.22), strongly calcareous, porous (45% porosity), and the organic matter content is very low (<0.5%). The saturation percentage of the soil is 26 and has a high drainage capacity. ICBA soil is classified as Typic Torripsamments, carbonatic, and hyperthermic (Shahid et al., 2014). Soil amendment provided by the company Koblenz compost organic fertilizer (manufactured by Tadweer waste Treatment LLC, Dubai, UAE), was supplemented to the top of the soil at a rate of 40 tons FW. ha^−1^ (at 85% moisture) to increase the soil water-holding capacity and to deliver some of the deficient and required nutrients, such as nitrogen (N), potassium (K), sulfur (S), and micro-nutrients. The field was irrigated with fresh water and fertilization with phosphorous was conducted 2 weeks before sowing using a single supply of 45 kg P_2_O_5_ ha^−1^. Granular urea nitrogen (N) fertilizer was applied once at a rate of 30 kg N ha^−1^, 3 weeks after planting. An application of NPK fertilizer (20-20-20) at a rate of 30 kg ha^−1^ was also given 6 weeks after planting by fertilization.

The 2,671 barley lines were sown in plots of one row of 2 m length each. Plots were randomized in an augmented design, and salt tolerant check line (58/1A) was sown every 50 plots. Two rows of a local barley cultivar (cv. Omani) were sown around the experimental area to reduce edge effects. Approximately 50 seeds per line were hand sown at 2 cm spacing and 1 cm depth per row. The space between rows was 25 cm as per the drip irrigation system design. As we are dealing with sandy soil with very poor water holding capacity and the evapotranspiration was very high, all plots were irrigated twice per day for 5 min each time. Each plot received 4.4 L water per day. The USDA barley core collection was evaluated for different traits related to agronomic performance and yield, but in this paper we will focus only on Na^+^ and K^+^ content in the flag leaf as an indice of salinity tolerance. Plants were irrigated with fresh water (<1 dS m^−1^), referred to hereafter as the control condition, or with saline ground water (≈23 dS m^−1^), referred to hereafter as saline condition. Saline plots were irrigated with ground water (≈23 dS m^−1^) for the entire growing period, starting from the third week after germination and until the physiological maturity of the spikes. The distribution of drippers was homogenous and the distance between drippers allowed the overlapping of wetting fronts. Flag leaf was harvested during the heading time from all lines from the control and saline conditions. The electrical conductivity of the saturated soil extract at harvesting time was 16.8 dS m^−1^. Na^+^ and K^+^ content was determined from samples of 1 g flag leaf digested with 2% nitric acid in ultrapure Milli Q water. After complete digestion, the filtrate was analyzed by the Inductively Coupled Plasma Emission Optical Spectrophotometer (ICP-OES, Perkin Elmer) to determine Na^+^ and K^+^ concentrations in the flag leaf.

### Genotyping of the USDA barley core collection

The USDA Barley core collection, a sub-sample of 2,671 lines chosen to represent the genotypic and phenotypic variability within the entire USDA collection of 30,000 accessions, was genotyped using the Illumina 9K single nucleotide polymorphism (SNP) array (Comadran et al., 2012). Briefly, this SNP array was constructed from a combination of the existing Barley Oligo Pool Assay (BOPA) SNPs (Close et al., 2009) and SNPs discovered from RNA-seq analyses as described by Comadran et al. (2012). To generate the called SNPs from the RNA-seq data, short-read data from 10 common cultivars (Barke, Betzes, Bowman, Derkado, Intro, Morex, Optic, Quench, Sergeant, and Tocada) were aligned onto the Harvest 35 reference sequences (available at http://harvest-web.org/hweb/mainmenu.wc). These putative SNPs were then further processed through quality control measures including removing minor alleles, selecting those SNPs with adequate read depth, and removing duplicate SNPs so that there was only a single SNP per unigene. From the initial 31,616 SNP candidates, 5,010 SNPs were selected for their coverage, information content, and technical performance. These SNPs were combined with the existing 2,832 BOPA SNPs to construct the Illumina 9K array. Of the 7,842 SNPs, 3,968 have been genetically mapped using 360 individuals from a F_6_ RIL population derived from the F_1_ progeny of a cross between Morex and Barke (complete information regarding the SNP assay development can be found in Comadran et al. (2012) and at http://bioinf.hutton.ac.uk/iselect/app/, verified 8 Aug. 2017. All genotypic data used in this work is available from The Triticeae Toolbox (https://triticeaetoolbox.org/, verified 8 Aug. 2017).

### Population structure and kinship analysis

Population structure was investigated using a Bayesian model-based approach of clustering implemented in STRUCTURE (Pritchard et al., 2000) to assign individuals to subpopulations. A clustering was run from *K* = 2 to *K* = 10 with 20 iterations with 10,000 burning period and 10,000 MCMC (Markov Chain Monte Carlo) for each value of K clusters and the logarithm of the data was estimated using an admixture model with correlated allele frequency. The optimal K was determined based on the likelihood model across 20 runs using the Evanno method (Evanno et al., 2005). The membership coefficient for the optimal K was permutated to match the various replicates for that value of K using CLUMPP (Jakobsson and Rosenberg, 2007). For visualization, we used the plotting function implemented in DISTRUCT software (Rosenberg, 2004). Using the method of Ritland (1996), we estimated kinship (K) using the software SPAGeDi (Hardy and Vekemans, 2002).

### Genome wide association analysis

The genome-wide association study was performed on the content of flag leaf sodium, potassium and their ratio using 3,968 genome-wide SNP markers. The association analysis was performed using the Genome Association and Prediction Integrated Tool package (GAPIT) (Zhang et al., 2010; Lipka et al., 2012), using the optimum compression mixed linear model and P3D options to increase speed and statistical power. To control for population structure and relatedness, the mixed model incorporated principal components (Price et al., 2006) and a kinship matrix (Ritland, 1996). The amount of phenotypic variation explained by the model was assessed using the R^2^ statistics. To correct for multiple testing problem, the procedure by Benjamini and Hochberg (1995) was used at a false discovery rates (FDRs) of 5%. A neighboring joining (NJ) tree was generated using the R package ape (Paradis et al., 2004). Principal component was plotted with the R package scatterplot3D (Ligges and Mächler, 2003). Linkage disequilibrium analysis was performed using HAPLOVIEW v.4.2 (Barrett et al., 2005) and the candidate genes located within and/or adjacent to the associated SNPs were identified using the website Barleymap (http://floresta.eead.csic.es/barleymap/) using the Morex genome annotation data.

### Evaluation of salinity tolerance of selected sensitive and tolerant lines in hydroponics

Based on the field trial screening of the USDA barley core collection for salinity tolerance and ICP for the estimation of sodium and potassium content in the flag leaf, our results showed that the maintenance of K^+^ with exclusion of Na^+^ from the flag leaf was highly correlated with salt tolerance. The relationship to discriminate between sodium and potassium and for which a simple index, the Na^+^/K^+^ ratio was strong enough to be exploited as a selection tool to screen for salinity tolerance. In fact, *Saltol* QTL was reported as a major association with Na^+^/K^+^ ratio measured at reproduction stage in mapping salinity tolerance in rice. Using this index, we selected five barley lines showing the lowest Na^+^/K^+^ ratio and accumulate less Na^+^ in the flag leaf, along with five sensitive lines for deep characterization of the maintenance of K^+^ acquisition with exclusion of Na^+^ from the flag leaf, which has been found highly correlated with plant salt tolerance in the field trial. The experiment was designed to conduct plant growth in hydroponic culture conditions onto half-strength Hoagland's solution (Davenport et al., 2005) under greenhouse conditions and in triplicate for each treatment. Salt treatment (0, 7, and 15 dS m^−1^) was applied at the early three-leaf stage and was maintained until the end of the plant growth cycle. Leaf, leaf sheath and root samples were harvested after 2 weeks of performing salt treatment for Na^+^ and K^+^ analysis. Scoring symptoms of survival, wilting, chlorosis, senescence, and death of the plants assessed salinity tolerance.

### Molecular cloning of the *HKT1;5* alleles

DNA was isolated from the flag leaf of the panel of 5 tolerant and 5 sensitive barely accessions, using the CTAB method. PCR amplification of the *HKT1;5* gene was accomplished with *Pfu* DNA polymerase (Promega) and specific primers (forward: 5′-CTAGCGCAGCTGTCGCTCTT-3′; and reverse: 5′-ACGTTGAAGTTGAGTGGGTC-3′). The PCR protocol consisted of an initial denaturation at 95°C for 3 min, followed by 30 cycles comprising a first step at 95°C for 30 S, an annealing step at 56°C for 30 S and an elongation step at 72°C for 3 min, and a final extension at 72°C for 5 min. Purified amplified products were cloned into the PCR cloning vector pSC-A-amp/kan from StrataClone, according to the manufacturer's protocol, and sequenced. The sequences were polished and aligned to each other, to other *Hordeum* and *Triticum* species.

### Real time PCR

Total RNA from 2 tolerant and 2 sensitive barely accessions under control and salt treatment (200 mM NaCl) was isolated with RNeasy plant mini kit (Qiagen). The remaining genomic DNA was removed by treating RNA with DNase-RNase free (Promega). The first strand cDNA was synthesized from 1 μg of total RNA, using the SuperScript kit (Invitrogen), according to the manufacturer's protocol.

Real-time PCR was performed in 384-well plates with the Light Cycler ® 480 Real-Time PCR System (Roche) using SYBR Green I (Roche). Oligonucleotides were designed using Primer 3. Primers were used for *HKT1;5* gene (forward, 5′-tcgtgcatagccatcttcgt-3′, and reverse 5′-GATGCTGAGGACGTTGAAGT-3′). Actin gene from barley was used as a housekeeping gene, to calculate a normalization factor. Primers used for the actin gene were (forward: 5′-CAATGTTCCTGCCATGTACG-3′, and reverse: 5′-ATGAGGAAGGGCGTATCCTT-3′). PCR reactions were performed in a 10 μl final volume, containing 3 μl cDNA (40 ng of cDNA), 0.5 μl of each primer (at 10 μM), 5 μL 2x SYBR Green I master mix and 1 μl of RNase-free water (Sigma). The reaction consisted of an initial denaturation at 94°C for 10 min followed by 45 cycles at 94°C for 10 s, 60°C for 10 s, and 72°C for 15 s. three biological repetitions were performed to calculate the expression level.

## Results

### Salinity tolerance of the USDA barley core collection

The barley core collection tested in the field exhibited significant difference in salinity tolerance. Traits were mapped at the heading stage for flag leaf length, width, weight, Na^+^ and K^+^ content, and at the maturity stage for grain yield. The scores were presented in Supplementary Figure 1 and show the density distribution of the phenotypic variation for the different traits measured.

**Figure 2:**
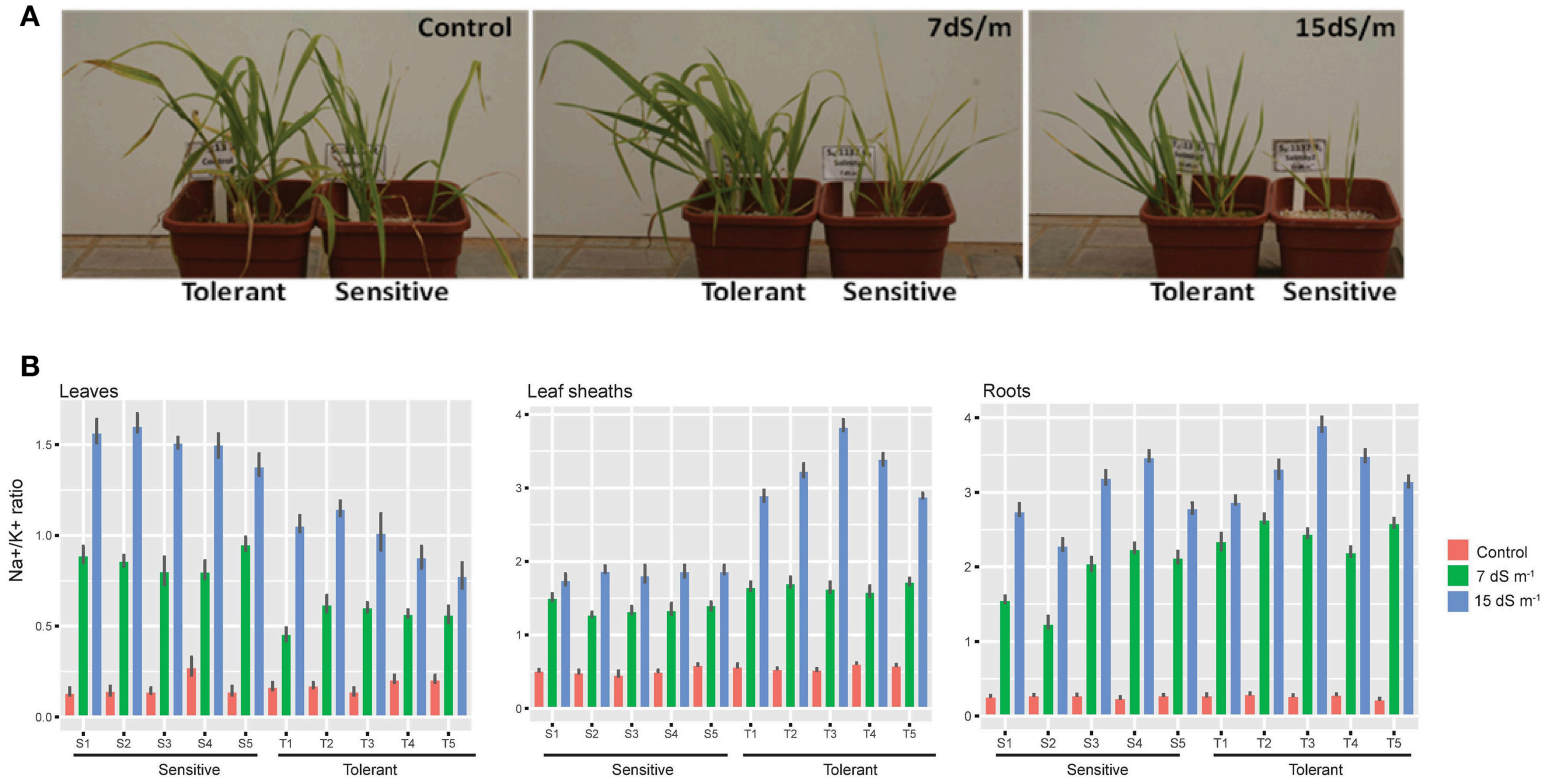
Na^+^ measures in different barely tissues. **(A)** A photograph of barely plants tolerant and sensitive subjected to Control, 7 and 15 ds m^−1^. **(B)** Five tolerant and five sensitive barely plants subjected to 7 and 15 ds m^−1^ compared to a control. Measures of Na^+^ in Leaf, leaf sheath and roots shown in barplot with mean ± standard error (*P* < 0.05), in salt tolerant and sensitive compared to control are shown.

Seeds from the five tolerant and five sensitive lines were sown in perlite and the experiment was designed to conduct plant growth in hydroponics system with half-strength Hoagland's solution under greenhouse conditions and in triplicate for each treatment. Salt treatment (0, 7, and 15 dS m^−1^) was applied at the stage of three leaves and was maintained until the end of the plant cycle. The sensitive lines exhibited severe symptoms of toxicity and senescence when plants are challenged with 15 dS m^−1^ of salt, while the tolerant lines exhibited moderate symptoms of toxicity and senescence (Figure 2A).

The tolerant and sensitive lines showed no significant difference in unidirectional root uptake of sodium under control and salt stress conditions at 7 and 15 dS m^−1^ (Figure 2B). However, the capacity of the leaf sheath in the tolerant lines to extract and sequester sodium as it entered the leaf was higher when the plants were challenged with salt stress. The sensitive lines were leaking sodium to the upper shoots and accumulate more sodium in their flag leaf. In contrast, the tolerant lines showed the lowest Na^+^/K^+^ ratio in their flag leaf, suggesting a lower rate of transfer of sodium from the roots to the shoots (Figure 2B). Statistical analysis was done using the Dunn (1964) Kruskal-Wallis multiple comparison with *p*-value adjusted with the false discovery rate method using an R package (see Supplementary Table 1). Our results are consistent with the finding of James et al. (2006), where the lower rates of net Na^+^ loading of the xylem is due to lower rates of Na^+^ transport from roots to shoots, and not to lower rates of net uptake of Na^+^ from the soil solution or higher rates of retranslocation in the phloem.

### Population structure

The population structure analysis assigned the genotypes to five subpopulations *K* = 5 with some admixture individuals in each subpopulation (Figure 1B), using a probability of membership *P* ≥ 0.8. This result is consistent with previous studies using the same USDA barley core collection (Muñoz-Amatriaín et al., 2014). At the contrary, this is not consistent with Fan et al. (2016), may be due to an arbitrary threshold of membership they consider in their analysis. Our structure analysis is consistent with the 3D principal Components analysis as well as the neighboring joining tree (NJ) that shows five clusters representing the five subpopulations (Figures 1C,D).

### Genome wide association analysis

**Figure 3:**
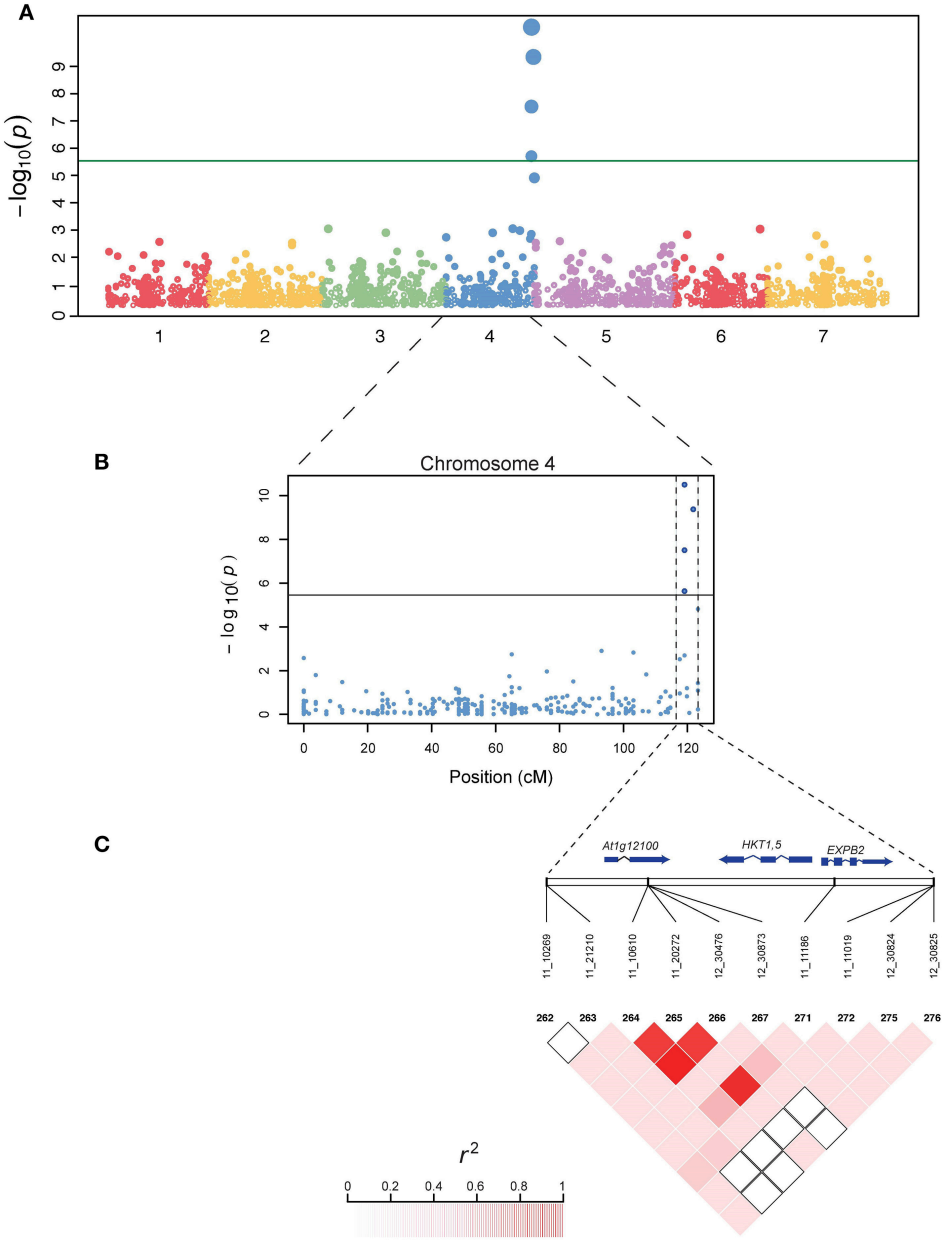
Genome wide association (GWAS) study in barley. **(A)** A Manhattan plot generated showing the seven barley chromosomes and the significant SNPs on the chromosome 4. The y-axis is the negative log_10_ transformed *p*-values of SNP from a genome-wide association analysis for Na^+^ plotted against the genetic distance in cM. **(B)** A zoom view of chromosome 4 with the bottom showing the candidate range for the gene *HKT1;5* associated with low Na+ accumulation in barley using the Morex annotation genome. **(C)** A panel depicting the extent of linkage disequilibrium in this region based on r^2^. The r^2^ values are indicated using color intensity at the left bottom. A region of 1.5 Mb associated with *HKT1;5* including other genes are indicated using two vertical dashed lines. Genes are represented in the middle panel.

The genetic basis of salt tolerance in *Hordeum vulgare* was examined using a unified mixed model that controls for population structures and kinship. At least four SNPs were most significant for the Na^+^ trait as well as the ratio K^+^/ Na^+^ at a genome-wide Bonferroni-corrected threshold that was estimated based on the effective number of independent tests (Table 1). No significant SNPs were found for the potassium K^+^ trait. Given the complex architecture of the salt tolerance trait, the most significant SNPs using this unified mixed model for both Na^+^ and K^+^/Na^+^ were located within 2 Mb region on the distal part of chromosome four (Figures 3A,B, Table 2). There are four SNPs that are significant (Table 1). Two of the SNPs (11_11186 and 11_20272) were significantly associated with the low accumulation of Na^+^ in the leaves. On average, the lines carrying the major frequency allele of the peak SNPs (11_11186 and 11_20272) were accumulating 15.5 mmoles more than with the alternative allele, and their effects were in the opposite direction. The other two SNPs (11_10610 and 12_30476) are associated with the trait and the allelic effect estimate is in the same direction (Supplementary Figure 2). The SNPs individually in both traits accounted from 16 to 28% of the total phenotypic variation elucidated by the traits and they are in linkage disequilibrium (LD) (*r*^2^ = 0.72–0.83) with each other (Figure 3C).

**Table 1:** Significant SNPs in Na^+^ and K^+^/Na^+^ ratio trait from the GWAS analysis.

**Na^+^ (SNPs)**	**Chromosome**	**Position**	***P*-values**	**MAF^a^**	**R^2^ (%)^b^**	**FDR_adjusted_Pvalues^c^**
11_20272	4	639755066	6.73E-22	0.274	18.2	1.56E-18
11_11186	4	639392844	2.44E-18	0.435	17.6	2.83E-15
11_10610	4	638202331	2.71E-15	0.435	17.12	2.10E-12
12_30476	4	638223459	1.53E-13	0.455	16.8	8.88E-11
K^+^/Na^+^ (SNPs)	4					
11_11186	4	639392844	1.29E-18	0.435	28.2	3.01E-15
11_20272	4	639755066	4.04E-18	0.274	28.2	4.69E-15
11_10610	4	638202331	3.67E-13	0.435	27.4	2.84E-10
12_30476	4	638223459	1.34E-11	0.455	27.2	7.78E-09

a Minor allele frequency

b percent phenotypic variation explained by the trait

c False discovery rate adjusted p-value

**Table 2:** Positions of the significant markers on the morex genome.

**Marker**	**Chr**	**Start**	**End**	**Strand**	**Other alignments**	**Gene_class**	**Description**	**InterPro**	**GeneOntologies**	**PFAM**
HORVU4Hr1G087760	Chr4H	638201783	638202868	+	No	HC_G	Bifunctional Inhibitor/lipid-transfer protein/seed storage 25 albumin superfamily protein	IPR016140 IPR027923		PF14547
11_10610	Chr4H	638202331	638202332	+	No					
HORVU4Hr1G087780	Chr4H	638223190	638275418	-	No	HC_G	Bifunctional Inhibitor/lipid transfer protein/seed storage 25 albumin superfamily protein	IPR016140 IPR027923		PF14547
HORVU4Hr1G087790	Chr4H	638223364	638224442	+	No	LC_u	Unpredicted protein			
12_30476	Chr4H	638223459	638223460	+	No					
HORVU4Hr1G087960	Chr4H	638634849	638636785	-	No					
HORVU4Hr1G087960	Chr4H	638634849	638636785	-	No	HC_G	Sodium transporter HKT1	IPR003445	GO: 0006812 GO: 0008324 GO: 0055085	PF02386
11_11186	Chr4H	639392844	639392845	+	No					
HORVU4Hr1G088140	Chr4H	639752735	639755477	+	No	HC_G	Expansin B2	IPR007118 IPR009009 IPR005795 IPR007112 IPR007117	GO: 0005576 GO: 0019953	PF01357 PF03330
11_20272	Chr4H	639755066	639755067	+	No					
HORVU4Hr1G089510	Chr4H	642560530	642564723	-	No	HC_G	Beta-amylase 5	IPR017853 IPR018238 IPR001371 IPR001554 IPR013781	GO: 0000272 GO: 0005975 GO: 0016161	PF01373
11_11019	Chr4H	642560940	642560941	+	No					

Annotation of the genes anchored close to those markers are summarized with emphasize on the HKT1;5 ge

### Co-localization of candidate genes associated with SNPs

Four significant SNPs were shown to be around an important *HKT 1;5* gene, which is a known major player in salt tolerance in wheat, rice and Arabidopsis. The SNP 12_30476 is around 0.5 Mb away from this transporter. There are multiple genes (Table 2) that are in the region that are part or adjacent to those significant SNPs. Genes contained in this region with significant SNPs association with reduce accumulation of Na^+^ from the Manhattan location also have some predicted functions such as lipid-transfer protein, flavin-containing monooxygenase family protein, zinc finger protein, Protein NRT1/ PTR FAMILY 6.3 and expansin B2. The ortholog of these genes was shown to play a role in salt tolerance in other species (Chen et al., 2012; Jülke and Ludwig-Müller, 2015; Kong et al., 2016; Zhang et al., 2016).

### Molecular cloning of the *HKT1;5* alleles

Isolation of the barley *HKT1;5* transporter gene from the tolerant and sensitive lines was attempted, considering the availability of the full length *HKT1;5* gene sequence in the Genbank (accession # DQ912169.1). The reference sequence was used to design primers for amplification by PCR of the barley *HKT1;5* genomic DNA. Eight alleles of the *HKT1;5* gene were amplified, cloned, sanger sequenced and aligned. A midpoint rooted phylogenetic tree showing the relationship between the different lines (tolerant and sensitive) along with the *HKT1;5* reference sequence for barley and other species was generated (Supplementary Figure 3). The assembled sequences from the tolerant and sensitive lines did not show any allelic variation that is linked to function. We could not find any nucleotide substitution in the coding region that differentiate the tolerant from the sensitive ones. Moreover, the analysis of cis-elements of 1 kb fragment from the *HKT1;5* promoter sequence (using http://bioinformatics.psb.ugent.be/webtools/plantcare/html/) gene between the tolerant (1_kb_PI138711.2_tolerant; 1_kb_PI21378_tolerant) and sensitive (1_kb_Chlo6091.2_sensitive; 1_kb_PI46735 8_sensitive) lines did not show any differences. A total of 28 cis-elements were distributed along the sequence (Supplementary Table 3). These cis-elements don't show any direct relation to regulation of genes under salt stress. However, we can't rule out that these cis-elements could modulate in a positive and indirect way the expression of *HKT1;5* gene in the leaf sheath of the tolerant compared to the sensitive lines. Further analysis is needed in this perspective.

### Real time PCR

**Figure 4:**
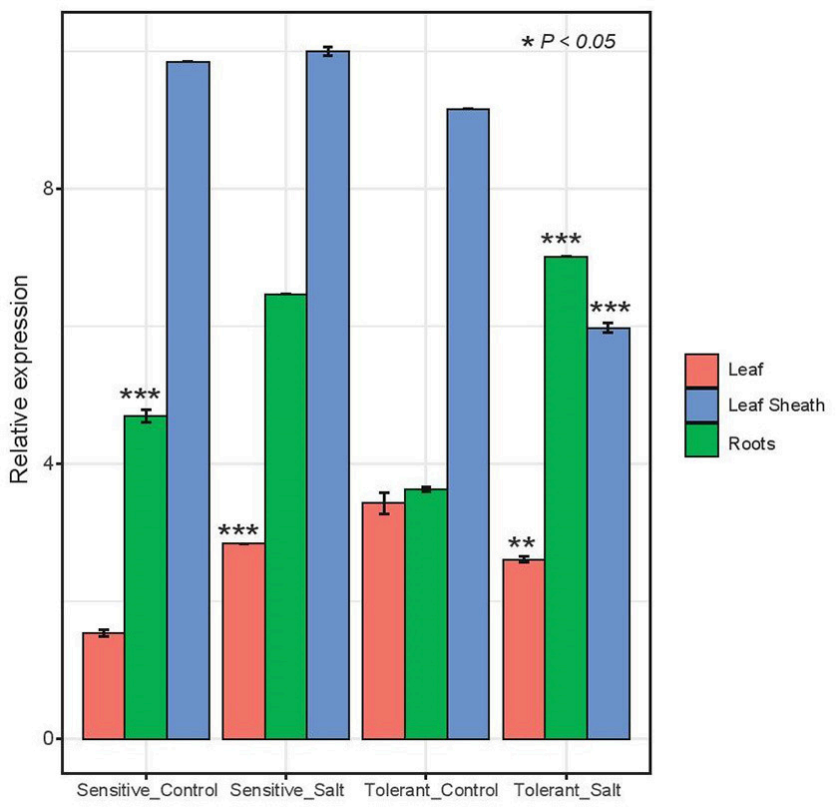
Expression analysis of HKT 1;5 by real time RT-PCR. Amplification of RNA from leaf, leaf sheath and root tissues of two barley salt tolerant and two salt sensitive lines. Sampling was done in triplicates after 48 h of growing the plants under salt stress condition (200 mM NaCl). A house keeping gene actin from barely was used for control and the data was normalized to 0 mM NaCl. The range of expression level (means ± SD, *n* = 3 biological replicates) in each line and tissue was analyzed with a *t*-student test and significance was reported (^*^P < 0.05; ^**^P < 0.005; ^***^P < 0.0005).

Expression of the *HKT 1;5* gene in the salt sensitive barley leaf compared to the control showed after 72 h of salt treatment a significant increase (*p* < 0.05) in the expression than the control plants that are not subjected to salt stress. In contrast, the salt tolerant barley leaf showed a significant decrease in expression (*p* < 0.05) than the control plants (Figure 4). Expression of the *HKT 1;5* gene in the salt sensitive barley roots as well as in the salt tolerant ones compared to the control showed a significant (*p* < 0.05) increase in the expression level (Figure 4). Expression of the *HKT 1;5* gene in the salt sensitive barley leaf sheaths compared to the control plants showed no significant increase in the expression. At high concentration of salt (200 mM NaCl), the reduction of the expression level of *HKT1;5* gene in leaf sheath of the tolerant line is correlated with increases in Na^+^ accumulation in this tissue. This is reflecting that *HKT1;5* gene play an important role in restricting the transport of Na^+^ from leaf sheath to the upper leaves. The range of expression level (means ± SD, *n* = 3 biological replicates) in each line and tissue was analyzed with a *t*-student test with reported *P*-value (< 0.05) (Supplementary Table 2).

## Discussion

Salinity tolerance involves a complex of responses at molecular, cellular and whole plant levels and is governed by the action of multiple genes that are highly affected by the environment, and genotype-by-environment (G × E) interactions (Arzani and Ashraf, 2016). Sodium exclusion play a major contributor of the mechanism conferring salt tolerance (Munns et al., 2006). Salt tolerance in plants was shown to depend on *HKT* transporters, which mediate Na^+^-specific transport or Na^+^- K^+^ co-transport and illustrate a key role in regulation of Na^+^ homeostasis (Rodríguez-Navarro and Rubio, 2006). For instance, a source of sodium exclusion named *Nax2*, found in the diploid ancestral wheat relative, *Triticum monococcum*, confers a decreased rate of Na^+^ transport from roots to shoots by retrieving Na^+^ from the root xylem into xylem parenchyma cells (Davenport et al., 2005; James et al., 2006). A candidate gene in the *Nax2* locus from *T. monococcum* (*TmHKT1;5-A)* and a major salt tolerance locus from bread wheat *Kna1* (*TaHKT1;5-D*), encode a Na^+^-selective transporter, expressed in stellar root cells surrounding xylem vessels, and therefore can limit the amount of Na^+^ that is translocated in the xylem to the leaf tissues (Munns et al., 2012; Byrt et al., 2014).

A new QTL for salinity tolerance in barley was identified through GWAS study (Fan et al., 2016). Association mapping for salinity tolerance was performed on 206 barley accessions and 408 DArT markers. Only two significant marker-trait associations for one QTL were detected on 4H. The authors showed that QTL on 4H with the nearest marker of bPb-9668 was the most significant, consistently detected in all methods. In the present study, using GWAS we mapped important SNPs that are located within 0.5 Mb from an important *HKT1;5* gene in barley, which may play a significant role in reducing Na+ concentration in the leaves. Since we don't have the sequence that expand this region in all the lines, more sequencing could be done across the majority of the barely accession (tolerant and sensitive) for better quantification. Other genes co-localizing in this region that have orthologs involved in salinity stress tolerance in other species could also play potential candidates for future validation. The application of linked SNP molecular markers for salinity tolerance in barley would be suitable to relevant crosses within the barley breeding program. They have the ability to cost effectively enable breeders to select germplasm and breeding lines using a simple DNA test without the need of a lot of phenotypic variation.

In rice and Arabidopsis, allelic variation for *OsHKT1;5* and *AtHKT1;5* was shown to be linked to the function of the gene (Ren et al., 2005; Rus et al., 2006). Our result in barley for 5 tolerant and 5 sensitive lines showed no allelic variation for the *HKT1;5* gene that is correlated with the phenotype, which is similar to what has been identified in wheat (Byrt et al., 2007). This result seems to suggest that difference in leaf Na^+^ concentration between the tolerant and sensitive lines, is mostly due to expression variation of this gene, rather than allelic variation.

The exclusion of Na^+^ is an important strategy employed by plants to prevent any shoot damage induced by Na^+^ accumulation. In barley, Na^+^ exclusion is an important salt tolerance strategy (Chen et al., 2007) as in wheat, however barley can tolerate more Na^+^ accumulated in the shoot than wheat (Munns and James, 2003; Colmer et al., 2005). This will suggest that tissue tolerance of Na^+^ in barley is a unique feature for salt tolerance. As an evidence of this, a recent study showed a constitutive overexpression of *HKT2;1* gene that increased salt tolerance in barley, showed an increase in the level of Na^+^ concentration in the shoots (Mian et al., 2011).

*HKT1;5* gene expression in the sensitive leaf sheath was the highest and did not change upon salt stress. However, Na^+^ accumulation in leaf sheath was much lower than in the tolerant line and there is no evidence of removal of Na^+^ from xylem in the leaf sheath of the sensitive line and Na^+^ is leaking to the upper shoots. In the tolerant line, *HKT1;5* gene expression was decreased upon salt treatment and Na^+^ accumulation in this tissue was higher than in the sensitive line, reducing its transport to the upper shoots. Similarly, reduced expression level of Na^+^ transporter, *TaHKT1;5* gene by gene silencing in transgenic bread wheat lines, increases Na^+^ accumulation in the leaves which reflecting that *TaHKT1;5* has in important role in restricting the transport of Na^+^ from the root to the leaves in bread wheat and showed the potentiality to increase salinity tolerance in bread wheat by the manipulation of *HKT1;5* gene (Byrt et al., 2014). In roots, there is an increase in the expression of *HKT1;5* gene in both tolerant and sensitive lines and no significant effect on root Na^+^ accumulation was observed. The precise control of Na^+^/K^+^ selective accumulation in leaf, leaf sheath and root tissues is an essential mechanism to maintain cellular homeostasis in the presence of imposed high salt concentration in the growth solution. It was shown in bread wheat that *TaHKT1;5* confers the essential salinity tolerance mechanism associated with the *Kna1* locus via shoot Na^+^ exclusion and is critical in maintaining a high K^+^/Na^+^ ratio in the leaves (Byrt et al., 2014). In rice, it was shown that *OsHKT1;5* plays a major role in the removal of Na^+^ from the xylem sap into the surrounding xylem parenchyma cells, thereby protecting leaves from Na^+^ toxicity (Ren et al., 2005). Possible roles of known HKT transporters in controlling Na^+^ flux in barley could function as an Na^+^ uptake system in the epidermal cortical cells and their expression could be down-regulated in conditions of salinity. This was shown to be the case for *OsHKT2;1*, which mediates the transport of Na^+^ into roots of K^+^-starved plants and was down-regulated when plants were exposed to salinity (Horie et al., 2007). In addition, comparative analysis using salt-tolerant and sensitive rice varieties have led to the hypothesis that *OsHKT1;4* restricts leaf-sheath-to blade Na^+^ transfer in rice plants under salinity stress (Cotsaftis et al., 2012). This mechanism is consistent with tissue specific expression of this gene that is regulating the transport of Na^+^ from root to leaves. In previous reports, it was demonstrated that *HKT* genes from *Arabidopsis thaliana* and rice, *AtHKT1;1* and *OsHKT1;5*, reduce transport of Na^+^ to the upper shoot and increase tolerance of the plant to salinity (Møller et al., 2009; Plett et al., 2010). Moreover, Munns et al. (2012) reported that *TmHKT1;5* encoding a Na^+^-selective transporter located on the plasma membrane of root cells surrounding xylem vessels, was perfectly localized to withdraw Na^+^ from the xylem and therefore reduce its transport to leaves. In accordance with the expression results, our data related to Na^+^ and K^+^ content analysis showed that the ratio Na^+^/K^+^ was associated with a higher root Na^+^ concentration in both tolerant and sensitive lines, while in leaf sheath there was a higher Na^+^ concentration than in leaf blade of the tolerant lines.

Our results support what has been known about *HKT 1;5* transporter gene and salt tolerance in barely, but it is the first time, that this gene is mapped using a GWAS approach. The question next is to test the function of this gene and its transport properties by performing electrophysiological recordings in Xenopus oocytes. We will design in the future an RNA interference experiment to knock down the gene in a tissue specific manner, study the transcript accumulation and see if the transgenic lines will accumulate more Na^+^ in their leaves. Similar work has been done in bread wheat, where *TaHKT1;5-D* expression was predominantly observed within the stele, particularly within xylem parenchyma and pericycle cells, which are adjacent to the xylem vessels, whereas *TaHKT1;5-D* transcripts was reduced in roots of transgenic lines containing an RNAi construct, increasing leaf Na^+^ concentration (Byrt et al., 2014).

Few data allowing to understand the different wheat and rice salt tolerance QTL linked to *HKT1;5* transporter genes are available so far. Indeed, the *Kna1* QTL involving the bread wheat D genome *TaHKT1;5* gene could certainly be explained by the low level of expression of the durum wheat *HKT1;5* genes as compared to *TaHKT1;5-D* (Byrt et al., 2014). The *Nax2* QTL, which has a similar mechanism to *Kna1* in bread wheat, involving *T. monococcum TmHKT1;5-A* gene might be similarly explained by higher expression of the *TmHKT1;5* gene as compared to durum wheat ones (Byrt et al., 2007, 2014; Munns et al., 2012). In rice, it was demonstrated that Na^+^ exclusion in the leaf was influenced by *OsHKT1;5* transcript abundance and that functional differences between the *OsHKT1;5* allele from salt-tolerant rice variety, Nona Bokra, and an allele from the salt-sensitive variety, Koshihikari, were referred to four amino acid substitutions (Ren et al., 2005; Cotsaftis et al., 2012). So far, 15 different *OsHKT1;5* alleles in rice showed a strong association with leaf Na^+^ concentrations (Negrão et al., 2013; Platten et al., 2013).

The present data of this work indicates that *HKT1;5* gene from barley has a higher expression in roots and therefore limiting the amount of Na^+^ transported in the xylem to the leaf tissues. Moreover, in previous work we demonstrated that alleles in the *Nax1* locus from durum wheat, *TdHKT1;4-1* and *TdHKT1;4-2* encode Na+ selective transporters and are effective in limiting Na^+^ accumulation in the leaves (Ben Amar et al., 2014).

## Conclusion

New insights into understanding the role of *HKT1;5* transporter gene in salt tolerance in barley was achieved. Using GWAS approach, we mapped for the first time *HKT1;5* gene from barley. Important SNPs markers were mapped and are located within 0.5 Mb from the *HKT1;5* gene and may play a significant role in reducing Na^+^ concentration in the leaves. Sequence analysis of *HKT1;5* alleles from tolerant and sensitive barley lines did not show any allelic variation that is linked to function and correlated with the phenotype. This is similar to what was found in wheat (Byrt et al., 2007). This seems to suggest that difference in leaf Na^+^ concentration between tolerant and sensitive lines, is mostly due to expression variation of *HKT1;5* gene, rather than allelic variation. The expression pattern of *HKT1;5* gene in barley revealed through real time PCR suggests that exclusion of Na^+^ from the roots is happening in the salt tolerant line due to the higher expression of the *HKT1;5* gene in the roots, but the accumulation of Na^+^ in the leaf sheath in the tolerant lines is consistent with the decrease in expression of *HKT1;5* gene in the leaf sheath and leaf to compensate the increase of expression in roots, which imply that the Na^+^ is going to accumulate less in the leaves of the tolerant lines than the sensitive. This mechanism is consistent with tissue specific expression of this gene that is regulating the exclusion of Na^+^ from the leaves. Ultimately, to study the function of *HKT1;5* gene in barley, knock down experiment via CRISPR-Cas9 technology or RNA interference will be applied in a tissue specific manner to study the accumulation of Na^+^ in leaves and the tolerance phenotype.

## Author contributions

KH, KA, and KM did the design of the paper and the analysis. BK did the real time PCR. DP and TB provided the SNP arrays and MS did the field testing. SM and DN did some laboratory work related to real time PCR. AM and KS-A discussed the manuscript. MP improved the manuscript.

### Conflict of interest statement

The authors declare that the research was conducted in the absence of any commercial or financial relationships that could be construed as a potential conflict of interest.

## Supplementary material

Supplementary Figure 1 Histogram plot of the phenotypic variation of the different traits (grain yield, average flag leaf width, average flag leaf length) measured. Wide dashed line represents the density distribution, while narrow ones represent the normal fitted distribution.

Supplementary Figure 2 Phenotypic differences between lines carrying different alleles of the four SNPs (11_10610; 12_30476; 11_11186; 11_20272) associated with salt concentration (Na^+^) showing allelic estimate and directionality. The boxplot shows the differences of Na^+^ concentration for the four SNPs showing the different alleles of the SNP locus. The box shows the first, second and third quartile. The width of the box is proportional to the square root of the number of individuals for each allele. The difference of mean (Δm) and the Pearson correlation (r) between the genotype and phenotype values as well as the P value of the correlation is shown on the side of the boxplots.

Supplementary Figure 3 A midpoint rooted phylogenetic tree showing the relationship between the different lines (tolerant and sensitive) along with the *HKT1;5* reference sequence for barley and other species was generated. The accession numbers are as follows: *Oryza_indica* (HQ162137.1), *Oryza_glaberrima* (JQ695813.1), *Oryza_japonica* (AP014957.1), *Oryza_rufipogon* (JQ695808.1), *Oryza_sativa* (JQ695818.1), *Triticum monococcum* (DQ646332.1), *Triticum_aestivum_cultivar Kharchia* (KU212875.1), *Hordeum_marinum_Subsp_marinum* (KF606928.1), *Hordeum_marinum_Subsp_gussoneanum* (KF606929.1), *Horedum_vulgare* (DQ912169.1).

Supplementary Table 1 Statistical analysis using the Dunn (1964) Kruskal-Wallis multiple comparison with *p*-value adjusted with the false discovery rate method for Na^+^/K^+^ ratio in roots, leaf sheaths and leaves at 0, 7, and 15 dSm^−1^.

Supplementary Table 2 Statistical analysis using the *t*-student test comparing the range of expression level (means ± SD, *n* = 3 biological replicates) in tolerant and sensitive lines and tissues (roots, sheaths and leaves) and *P*-value was reported (<0.05).

Supplementary Table 3 A summary of the putative 28 cis-elements along the 1 Kb sequence promoter of the *HKT1;5* gene in two tolerant and two sensitive lines using the PlantCARE database.
