# Supplementary figures and images for: Mapping of HKT1;5 Gene in Barley Using GWAS Approach and Its Implication in Salt Tolerance Mechanism
Source: Front Plant Sci. 2018 Feb 19;9:156. doi: 10.3389/fpls.2018.00156 (PMC5826053; doi:10.3389/fpls.2018.00156)

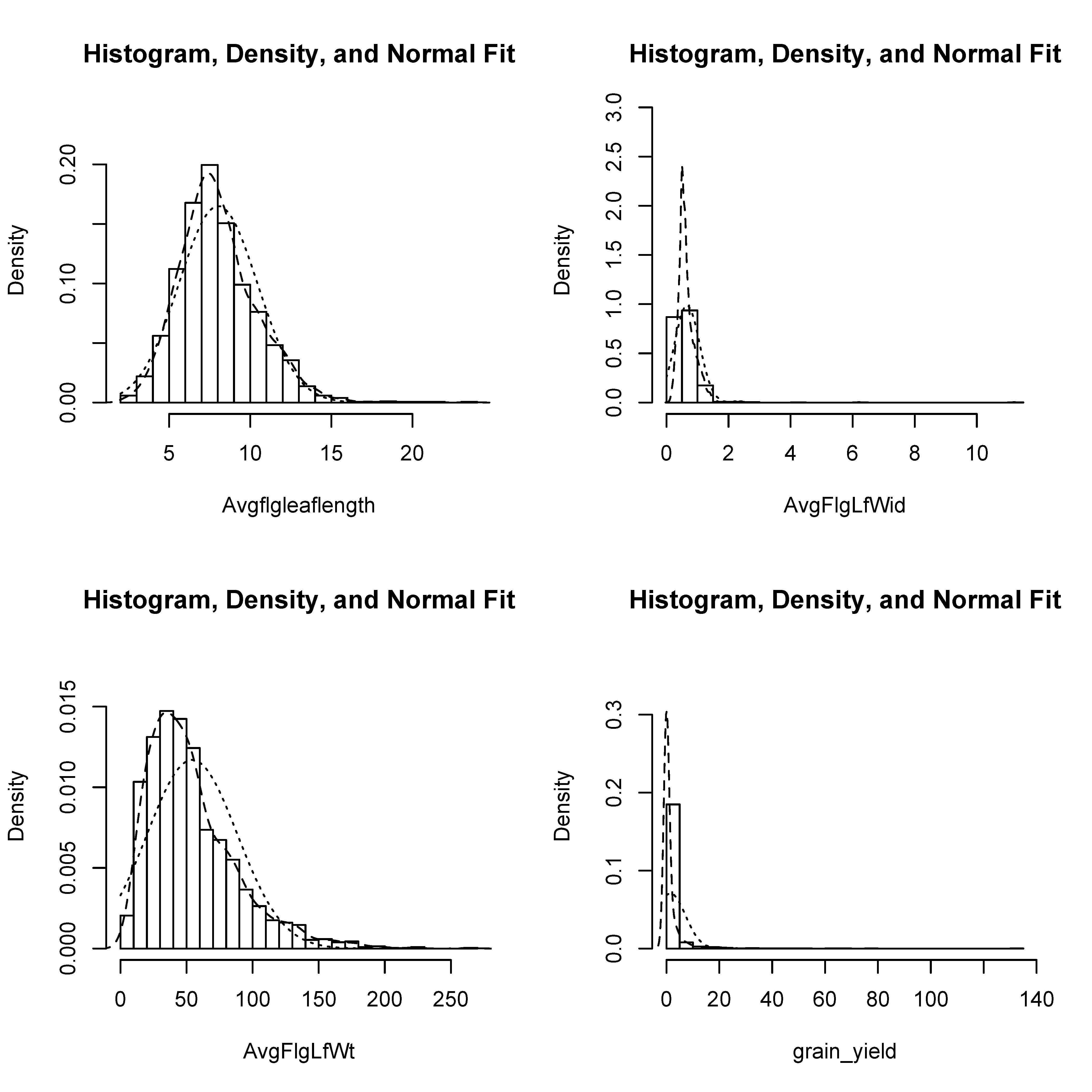

Supplement: Supplementary Figure 1 — Histogram plot of the phenotypic variation of the different traits (grain yield, average flag leaf width, average flag leaf length) measured. Wide dashed line represents the density distribution, while narrow ones represent the normal fitted distribution. [file Image1.JPEG]

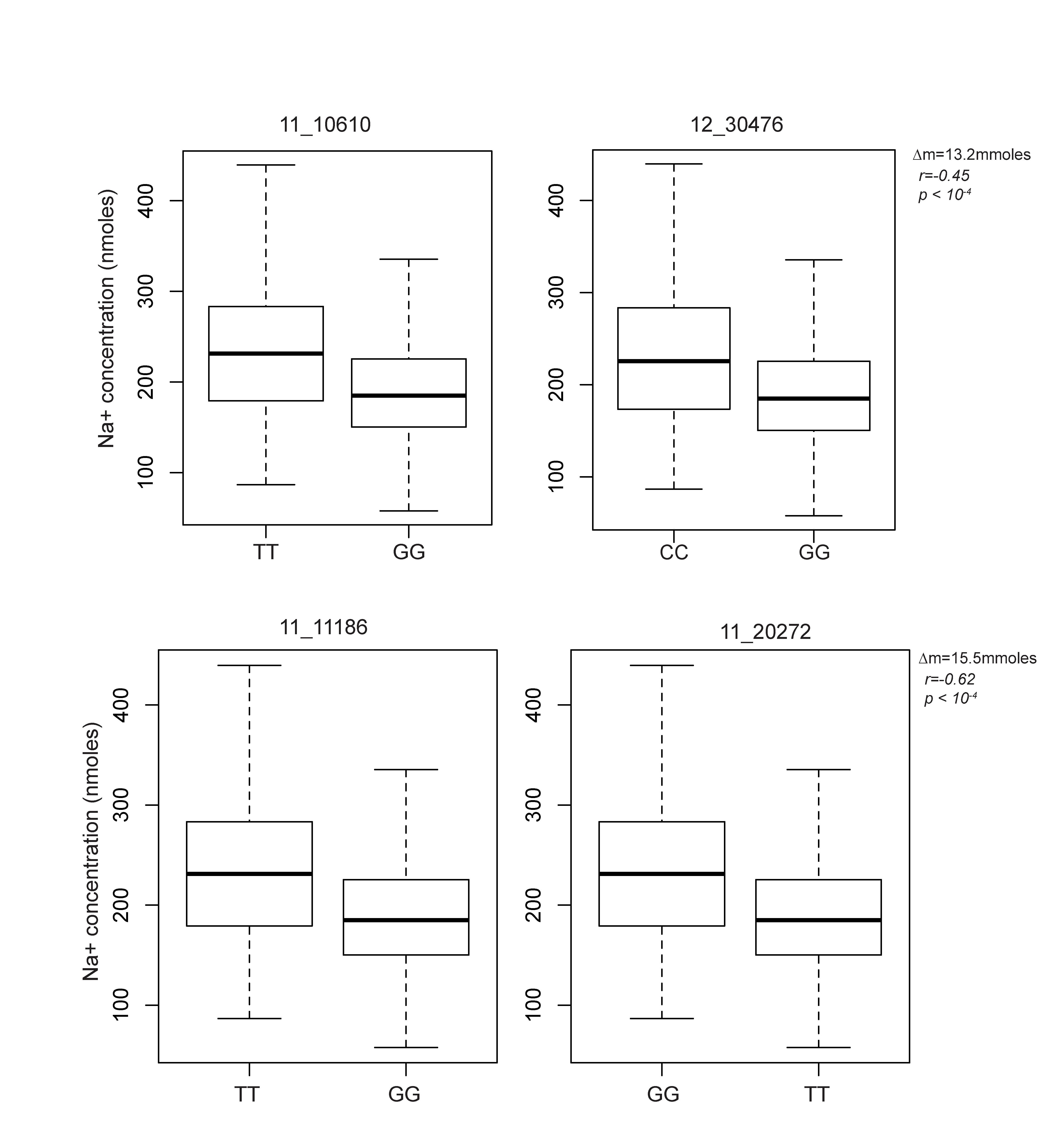

Supplement: Supplementary Figure 2 — Phenotypic differences between lines carrying different alleles of the four SNPs (11_10610; 12_30476; 11_11186; 11_20272) associated with salt concentration (Na+) showing allelic estimate and directionality. The boxplot shows the differences of Na+ concentration for the four SNPs showing the different alleles of the SNP locus. The box shows the first, second and third quartile. The width of the box is proportional to the square root of the number of individuals for each allele. The difference of mean (Δm) and the Pearson correlation (r) between the genotype and phenotype values as well as the P value of the correlation is shown on the side of the boxplots. [file Image2.JPEG]

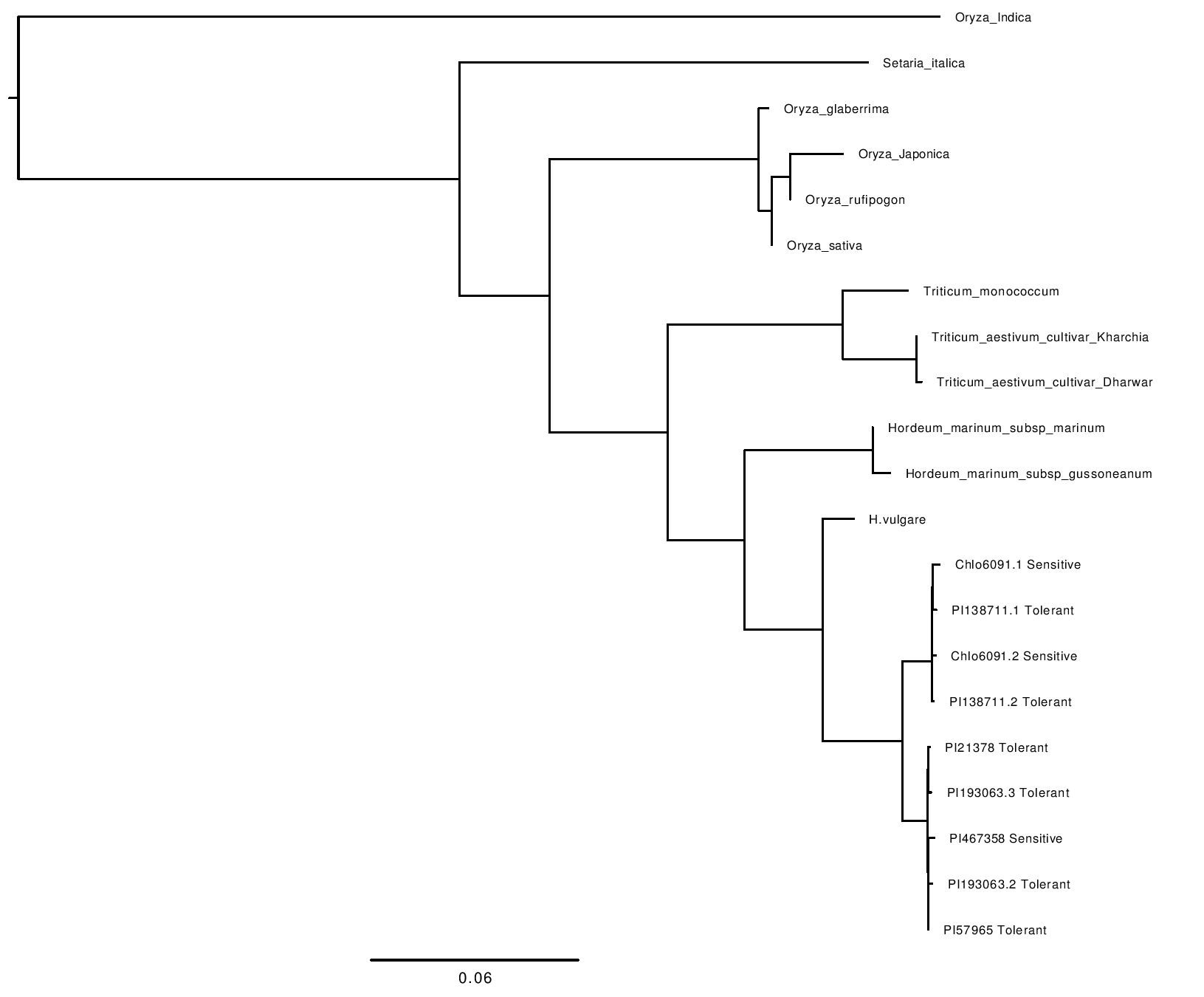

Supplement: Supplementary Figure 3 — A midpoint rooted phylogenetic tree showing the relationship between the different lines (tolerant and sensitive) along with the HKT1;5 reference sequence for barley and other species was generated. The accession numbers are as follows: Oryza_indica (HQ162137.1), Oryza_glaberrima (JQ695813.1), Oryza_japonica (AP014957.1), Oryza_rufipogon (JQ695808.1), Oryza_sativa (JQ695818.1), Triticum monococcum (DQ646332.1), Triticum_aestivum_cultivar Kharchia (KU212875.1), Hordeum_marinum_Subsp_marinum (KF606928.1), Hordeum_marinum_Subsp_gussoneanum (KF606929.1), Horedum_vulgare (DQ912169.1). [file Image3.JPEG]
